# Enhanced Somatic Embryo Induction of a Tree Peony, *Paeonia ostii* ‘Fengdan’, by a Combination of 6-benzylaminopurine (BA) and 1-naphthylacetic Acid (NAA)

**DOI:** 10.3390/plants9010003

**Published:** 2019-12-18

**Authors:** Xiuxia Ren, Ya Liu, Byoung Ryong Jeong

**Affiliations:** 1 Division of Applied Life Science (BK21 Plus Program), Graduate School of Gyeongsang National University, Jinju 52828, Korea; xiuxia0823@163.com (X.R.); liuya113@mails.ucas.ac.cn (Y.L.); 2 Institute of Agriculture and Life Science, Gyeongsang National University, Jinju 52828, Korea; 3 Research Institute of Life Sciences, Gyeongsang National University, Jinju 52828, Korea

**Keywords:** *Paeonia ostii* ‘Fengdan’, tree peony, somatic embryogenesis, development

## Abstract

Somatic embryogenesis is a preferred method for vegetative propagation due to its high propagation efficiency. In this study, zygotic embryos, cotyledons, and hypocotyls of *Paeonia ostii* ‘Fengdan’ were used as the explant to induce somatic embryogenesis. The results showed that a combination of 0.5 mg·L^−1^ thidiazuron (TDZ) and 0.5 mg·L^−1^ 2,4-dichlorophenoxyacetic acid (2,4-D) was effective in inducing somatic embryos from the zygotic embryo and cotyledon explants. Hypocotyls only formed somatic embryos on Murashige and Skoog (MS) medium supplemented with both 0.5 mg·L^−1^ TDZ and 0.5 mg·L^−1^ 1-naphthylacetic acid (NAA). Moreover, the compact callus was effectively produced from zygotic embryo, cotyledon, and hypocotyl explants in medium supplemented with a combination of 3.0 mg·L^−1^ 6-benzylaminopurine (BA) and 1.0 mg·L^−1^ NAA, and then converted into somatic embryos in the same medium, and the ratio of the explants with embryo induction and number of embryos induced per explant were much higher than those induced by 0.5 mg·L^−1^ TDZ and either 0.5 mg·L^−1^ 2,4-D or 0.5 mg·L^−1^ NAA. The MS medium was better than the woody plant medium (WPM) for inducing somatic embryos from zygotic embryo and hypocotyl explants, whereas the WPM was better than the MS medium for somatic embryogenesis induction from cotyledon explants. All of the somatic embryos developed well into mature embryos on their respective media supplemented with both 3.0 mg·L^−1^ BA and 1.0 mg·L^−1^ NAA. Overall, the protocols for indirect somatic embryogenesis from zygotic embryo, cotyledon, and hypocotyl of *P. ostii* ‘Fengdan’ were successfully established, which can greatly facilitate their propagation and breeding processes.

## 1. Introduction

*Paeonia ostii* ‘Fengdan’ is a wild tree peony species that belongs to Sect. Moutan DC., genus *Paeonia*, Paeoniaceae [1]. It is a well-known ornamental plant with perennial woody shrubs and large, ornate flowers, with more than 1500 years of history being cultivated [2]. It is also a famous medicinal plant known for its root bark, also known as cortexmoutan, which is an important ingredient in traditional Oriental medicine [1]. Furthermore, seeds of *P. ostii* ‘Fengdan’ have recently attracted much attention, since it has a high oil content (27%) with more than 90% unsaturated fatty acids and more than 40% α-linolenic acid content, which are especially beneficial to human health [3]. The commercialization of cortexmoutan, peony seed oil, and other processed peony products causes an ever-increasing demand for *P. ostii* ‘Fengdan’ plants. A large number of *P. ostii* ‘Fengdan’ plants need to be propagated rapidly in a short time. Traditionally, tree peony is propagated by seeds, division, and grafting, which are slow and time-consuming methods [4]. Therefore, asexual micropropagation is highly appreciated for its unique superiority.

Somatic embryogenesis is a technique in which the somatic cells of a mother plant develop into a whole new plant [5]. The whole process of somatic embryogenesis is very similar with zygotic embryogenesis [5], and the morphology of somatic embryos is similar to that of zygotic embryos [6,7]. It is a well-received technology for mass multiplication due to its high multiplication efficiency and short production time [8,9]. Somatic embryogenesis has been already developed in many horticultural crops [10,11,12,13,14]. Moreover, embryogenic cultures are also the most suitable assistive technology for genetic transformation. Conclusively, somatic embryogenesis is an excellent asexual reproduction method.

A feasible and optimal protocol for the somatic embryogenesis of *P. ostii* ‘Fengdan’ is still not yet available, even though some research has been done [15]. The aim of this study is to develop efficient plant regeneration protocols via somatic embryogenesis using zygotic embryos, hypocotyls, and cotyledons as the explants. The developed protocols will be helpful for the germplasm conservation, plant regeneration, and genetic improvement of tree peony.

## 2. Materials and Methods

### 2.1. Plant Materials and Disinfection

Disease-free mature seeds of *P. ostii* ‘Fengdan’ were thoroughly washed in running tap water for 12 h and subsequently dipped in 200 mg·L^−1^ gibberellin 3 (GA_3_) for 18 h. The pretreated seeds were disinfected using 70% ethanol for 5 min and 3% sodium hypochlorite (NaClO) for 5 min, and then rinsed with sterilized deionized water more than five times. Zygotic embryos were picked out from the sterilized seeds. A part of the zygotic embryos was cultured on Murashige and Skoog (MS) medium [16] supplemented with both 0.5 mg·L^−1^ 6-benzylaminopurine (BA) and 1.0 mg·L^−1^ GA_3_ for 4 weeks to produce hypocotyls and cotyledons in dark condition. The rest of the zygotic embryos, as well as the produced hypocotyls and cotyledons, were used as the explants for the following experiments.

### 2.2. The Effects of the Basal Medium and Different Plant Growth Regulator (PGR) Combinations on the Induction of Somatic Embryos

Combinations of two cytokinins (BA and thidiazuron, TDZ) and two auxins (1-naphthylacetic acid (NAA) and 2,4-dichlorophenoxyacetic acid (2,4-D)) at 0.5 mg L^−1^ each were added to the MS medium or woody plant medium (WPM) [17] containing 3% (*w/v*) sucrose and 0.80% (*w/v*) agar, as summarized in Table 1. The medium pH was adjusted to 5.80 before agar was added. All media were autoclaved at 121 °C for 15 min. The whole zygotic embryos (with 3.0 mm length and 1.5 mm width), cotyledons (around 5.0 mm in length), and hypocotyls (around 5.0 mm in length) were used as the explants in inducing somatic embryos. All explants were inoculated on these media under a dark, poikilothermic (24 °C for 16 h and 18 °C for 8 h) condition. The ratio of explants with embryo induction was recorded after 3 months of culture. Regular subcultures were carried out monthly, and the culture condition and time for data collection and subculture in 2.3 and 2.4 were same with that of 2.2.

### 2.3. The Effects of the Concentration Ratio of Cytokinin and Auxin on the Induction of Somatic Embryos

One kind of cytokinin (BA) and one kind of auxin (NAA) were used to study the effect of the concentration ratio of cytokinin and auxin on the induction of somatic embryos, which was based on the somatic embryo study in herbaceous peony [18]. A combination of BA (1.0, 2.0, 3.0, 4.0, and 5.0 mg·L^−1^) and NAA (1.0 mg·L^−1^) was added to the MS medium and WPM containing 3% (*w/v*) sucrose and 0.80% (*w/v*) agar.

### 2.4. The Effects of the Basal Medium and Explant Type on the Induction of Somatic Embryos 

The zygotic embryos, cotyledons, and hypocotyls were used as the explants in inducing the somatic embryos. A combination of 3.0 mg·L^−1^ BA and 1.0 mg·L^−1^ NAA was added to the MS medium or WPM containing 3% (*w/v*) sucrose and 0.80% (*w/v*) agar.

### 2.5. Morphological Observation of the Induction and Development of Somatic Embryos from the Zygotic Embryo

The zygotic embryos were cultured in MS medium supplemented with 3.0 mg·L^−1^ BA and 1.0 mg·L^−1^ NAA for 4 months under a dark, poikilothermic (24 °C for 16 h and 18 °C for 8 h) condition. Regular subcultures were carried out monthly. Morphological observation of the induction and development of somatic embryo was performed weekly by a stereoscopic microscope (Leica, Bensheim, Germany).

### 2.6. Statistical Analysis

Three replications with 20 explants per experiment were carried out in all experiments. Results were presented as means ± the standard error and analyzed using one-way ANOVA, followed by Duncan’s multiple-range test.

## 3. Results

### 3.1. Callus and Somatic Embryo Induction as Affected by Basal Medium, PGR Combination, and the Concentration Ratio of Cytokinin and Auxin

**Figure 1:**
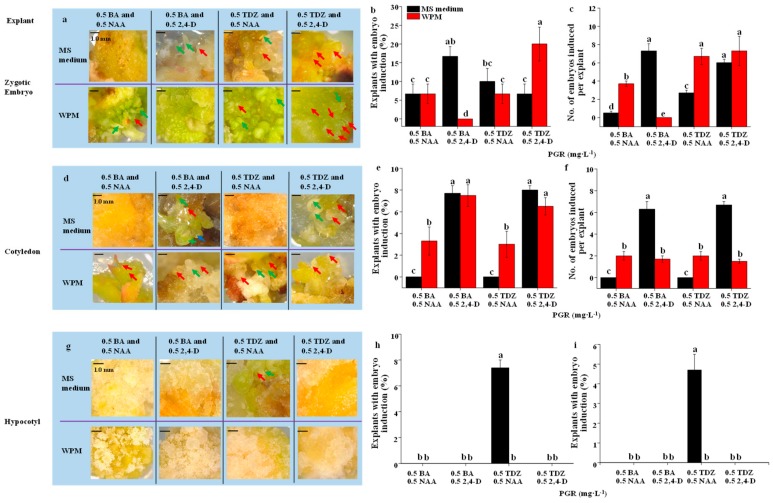
Effect of the basal medium and plant growth regulator (PGR) on morphology (**a**,**d**,**g**), ratio of the explants with embryo induction (**b**,**e**,**h**), and number of embryos induced per explant (**c**,**f**,**i**) from the zygotic embryo (**a**–**c**), cotyledon (**d**–**f**), and hypocotyl (**g**–**i**) explants. Scale bar is 1.0 mm. Red arrow represents somatic embryos in globular stage, green arrow represents somatic embryos in torpedo stage, and blue arrow represents somatic embryos in cotyledonary stage. The data were collected 3 months later. Different letters mean significant differences according to Duncan’s multiple range test at *p* ≤ 0.05. MS, Murashige and Skoog; WPM, woody plant medium.

Somatic embryos were successfully induced from the zygotic embryos, which were greatly affected by the combination of the basal medium and plant growth regulator (PGR). The somatic embryos in different developmental stages are shown in Figure 1a,d,g. The whole number of embryos in all developmental stages was recorded after 3 months of culture to determine the ratio of the explants with embryo induction and the number of embryos induced per explant. The ratio of explants with embryos induced from the zygotic embryos was the greatest on the WPM supplemented with both 0.5 mg·L^−1^ TDZ and 0.5 mg·L^−1^ 2,4-D in up to 20%, followed by the MS medium supplemented with both 0.5 mg·L^−1^ BA and 0.5 mg·L^−1^ 2,4-D (Figure 1a,b). The number of induced embryos per zygotic embryo was enhanced on the MS medium supplemented with both 0.5 mg·L^−1^ 2,4-D and either 0.5 mg·L^−1^ BA or 0.5 mg·L^−1^ TDZ, or on the WPM supplemented with both 0.5 mg·L^−1^ TDZ and either 0.5 mg·L^−1^ 2,4-D or 0.5 mg·L^−1^ NAA (Figure 1c) in up to 7. The ratio of explants with embryos induced from the cotyledon explants was the highly increased on either the MS medium or WPM supplemented with BA (0.5 mg·L^−1^) and 2,4-D (0.5 mg·L^−1^), or with TDZ (0.5 mg·L^−1^) and 2,4-D (0.5 mg·L^−1^) (Figure 1d,e), and the ratio of explants with embryo induction in above treatments was lower than 10%. Moreover, somatic embryos could also be induced from the cotyledon explants on the WPM supplemented with both 0.5 mg·L^−1^ NAA and either 0.5 mg·L^−1^ BA or 0.5 mg·L^−1^ TDZ (Figure 1d). The number of embryos induced per cotyledon explant was the greatest on the MS medium supplemented with both 0.5 mg·L^−1^ 2,4-D and either 0.5 mg·L^−1^ BA or 0.5 mg·L^−1^ TDZ (in up to 6), followed by the WPM supplemented with both 0.5 mg·L^−1^ BA and either 0.5 mg·L^−1^ NAA or 0.5 mg·L^−1^ 2,4-D, or both 0.5 mg·L^−1^ TDZ and either 0.5 mg·L^−1^ NAA or 0.5 mg·L^−1^ 2,4-D (Figure 1f). Hypocotyls only formed somatic embryos on the MS medium supplemented with both 0.5 mg·L^−1^ TDZ and 0.5 mg·L^−1^ NAA (Figure 1g). The ratio of explants with embryos induced from the hypocotyl explants was about 8% and the number of embryos induced per hypocotyl explant was around 5 (Figure 1h,i). Conclusively, the ratio of explants with embryo induction and the number of embryos induced per explant was the greatest in embryo, followed by cotyledon explant, and they were the lowest in hypocotyl explant.

**Figure 2:**
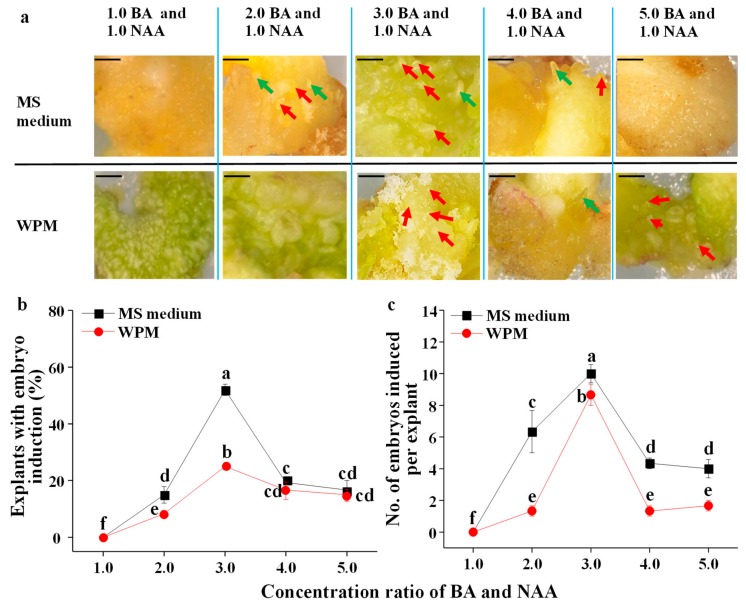
The effects of the basal medium and BA/NAA concentration on the morphology (**a**), ratio of the explants with embryo induction (**b**), and number of embryos induced per explant (**c**) from calli using the zygotic embryos as the explant. The unit of PGR (in Figure 2a) is in milligrams per liter (mg·L^−1^). Scale bar is 1.0 mm. Red arrow represents somatic embryos in globular stage, green arrow represents somatic embryos in torpedo stage, and blue arrow represents somatic embryos in cotyledonary stage. Different letters indicate significant differences according to Duncan’s multiple range test at *p* ≤ 0.05.

A combination of BA and NAA was effective in inducing callus (Figure S1), and from this, callus somatic embryos developed (Figure 2), which was greatly affected by medium and the ratio of BA and NAA concentrations (Figure 2a). The somatic embryos in different developmental stages are shown in Figure 2a. The whole number of embryos in all developmental stages was recorded after 3 months of culture to determine the ratio of the explants with embryo induction and the number of embryos induced per explant. The ratio of explants with embryo induction increased with the increase in the ratio of BA and NAA concentrations on both the MS medium and the WPM, then decreased with the further increase in the ratio of BA and NAA concentrations beyond 3:1 (BA/NAA) (Figure 2b). The ratio of explants with embryo induction was the greatest in MS medium supplemented with both 3.0 mg·L^−1^ BA and 1.0 mg·L^−1^ NAA (about 50%), followed by the WPM supplemented with both 3.0 mg·L^−1^ BA and 1.0 mg·L^−1^ NAA (about 25%) (Figure 2b), which was higher than those induced by both 0.5 mg·L^−1^ TDZ and either 0.5 mg·L^−1^ 2,4-D or 0.5 mg·L^−1^ NAA (the best result in the first experiment) (Figure 1). The ratio of explants with embryo induction was significantly greater in MS medium than that in WPM when the ratio of BA and NAA concentrations was 2.0–3.0. The number of embryos induced per explant exhibited similar trends to the ratio of embryo induction: It increased with the increase in the ratio of BA and NAA concentrations on both the MS medium and the WPM, then decreased with the increase in the ratio of BA and NAA concentrations beyond 3:1 (BA/NAA). The greatest number of embryos induced per explant was found in MS medium supplemented with both 3.0 mg·L^−1^ BA and 1.0 mg·L^−1^ NAA (10), followed by WPM supplemented with both 3.0 mg·L^−1^ BA and 1.0 mg·L^−1^ NAA (9) (Figure 2c), which was greater than those induced in the MS medium supplemented with both 0.5 mg·L^−1^ 2,4-D and either 0.5 mg·L^−1^ BA or 0.5 mg·L^−1^ TDZ, or on the WPM supplemented with both 0.5 mg·L^−1^ TDZ and either 0.5 mg·L^−1^ 2,4-D or 0.5 mg·L^−1^ NAA (the best results in the first experiment) (Figure 1c)—and it was significantly greater in MS medium than that in WPM when ratio of BA and NAA concentrations was 2.0–5.0.

### 3.2. Callus and Somatic Embryo Induction as Affected by the Basal Medium and Explant Type 

**Figure 3:**
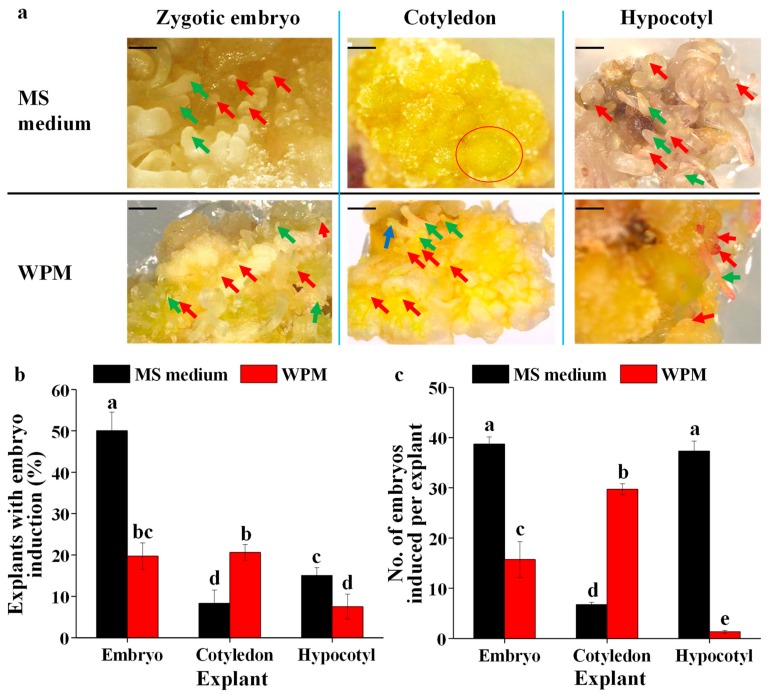
The effects of the basal medium and explant type on the morphology (**a**), ratio of explants with embryo induction (**b**), and number of embryos induced per explant (**c**). The scale bar indicates 1.0 mm. Red circle represents embryogenic callus, which is ready to develop into globular somatic embryos. Red arrow represents somatic embryos in globular stage, green arrow represents somatic embryos in torpedo stage, and blue arrow represents somatic embryos in cotyledonary stage. Different letters indicate significant differences according to Duncan’s multiple range test at *p* ≤ 0.05.

Callus were produced first in MS medium and WPM supplemented with 3.0 mg·L^−1^ BA and 1.0 mg·L^−1^ NAA (Figure S2), and then somatic embryos developed (Figure 3a). The percentage of the explant with callus induction induced from zygotic embryo and hypocotyl explants was significantly greater than that induced from cotyledon explants and there was no effect of basal medium (Table 1).

The somatic embryos in different developmental stages are shown in Figure 3a. The whole number of embryos in all developmental stages were recorded after 3 months of culture to determine the ratio of the explants with embryo induction and the number of embryos induced per explant. The MS medium was better for inducing somatic embryos from callus explants from zygotic embryos and hypocotyls, whereas the WPM was better for inducing somatic embryos from callus explants from cotyledons (Figure 3a). The ratio of explants with embryos induced from the zygotic embryos was the greatest on the MS medium, followed by that with embryos induced from the cotyledon explants cultured on the WPM, that with embryos induced from the zygotic embryos cultured on the WPM, and that with embryos induced from the hypocotyl explants cultured on the MS medium (Figure 3b). The number of embryos induced per explant was significantly higher than others from the embryo and hypocotyl explants on the MS medium, followed by that from cotyledon explants cultured on the WPM (Figure 3c).

### 3.3. The Development of Somatic Embryos

**Figure 4:**
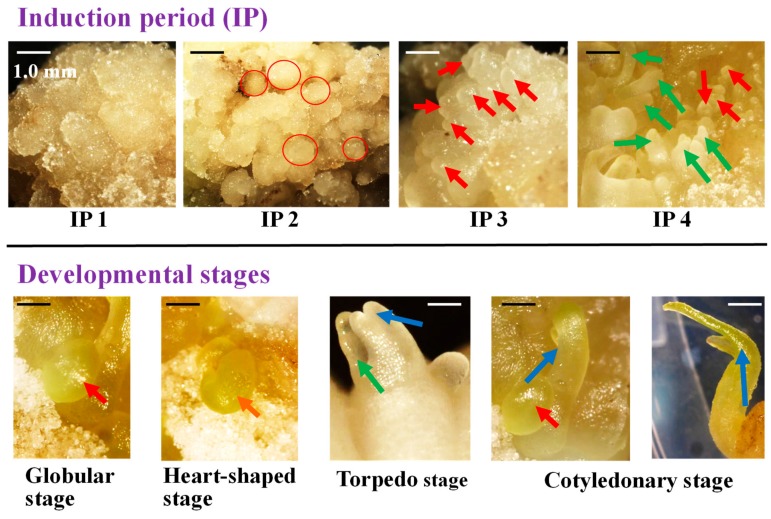
The morphology of somatic embryos in the different induction periods and developmental stages on the MS medium containing both NAA 3.0 mg·L^−1^ BA and 1.0 mg·L^−1^ NAA. The scale bar indicates 1.0 mm in IP 1–IP 4, while the scale bar indicates 0.2 mm in developmental stages. Red circle represents embryogenic callus, which is ready to develop into globular somatic embryos. Red arrow represents somatic embryos in globular stage, green arrow represents somatic embryos in torpedo stage, and blue arrow represents somatic embryos in cotyledonary stage.

The induced somatic embryos developed well into mature embryos on their respective media supplemented with both 3.0 mg·L^−1^ BA and 1.0 mg·L^−1^ NAA (Figure 4). Morphology of different induction periods and developmental stages from *P. ostii* ‘Fengdan’ somatic embryos was detected on the MS medium supplemented with both 3.0 mg·L^−1^ BA and 1.0 mg·L^−1^ NAA (Figure 4). Callus was induced first (Figure S2 and IP1 in Figure 4) after 1 month of culture. The callus was similar to normal callus (IP1 in Figure 4) at the very beginning (around 2–4 weeks after culture), and then changed to embryogenic callus with globular shape, roughness surface, and soft texture (IP2 in Figure 4) after 6–8 weeks of culture. After that, it changed to globular ball with smooth surface and compact structure after 8–12 weeks of culture, and these structures were somatic embryos in globular stage (IP 3 in Figure 4). Then, those somatic embryos developed into mature embryos in the same medium after 12–16 weeks of culture (IP4 in Figure 4). Developmental stages, including globular stage (IP 3), heart-shaped stage (IP 4), torpedo stage (IP 4), and cotyledonary stage (IP 4), are listed in Figure 4. Most of the time, somatic embryos in different developmental stages were always found at the same time, shown in IP 4 in Figure 4.

## 4. Discussion

As *P. ostii* ‘Fengdan’ is an important ornamental plant with great medicinal values, the establishment of an efficient micropropagation protocol deserves considerable attention. Somatic embryogenesis is a powerful tool in plant propagation with a high regeneration potential and low frequency of mutations [9]. Therefore, the effects of basal medium, PGR combination, concentration ratio of cytokinin and auxin, and the explant were analyzed in this study to build up efficient protocols for somatic embryogenesis of *P. ostii* ‘Fengdan’.

Hormones could regulate cell dedifferentiation and the initiation of embryogenesis [19,20,21,22]. Our results show that a combination of cytokinin (BA or TDZ) and auxin (2,4-D) was effective in inducing somatic embryos in *P. ostii* ‘Fengdan’. A combination of cytokinin (BA) and auxin (IBA) could also enhance somatic embryo induction from buds of Iton peony [23]. Numerous studies on the somatic embryogenesis in a wide range of species show that auxins have a critical role in the somatic embryo induction and cytokinins are also frequently involved [21]. A combination of auxins and cytokinins also induces somatic embryogenesis in *Medicago truncatula* [24]. It was found that auxins are critical for somatic embryogenesis in *Arabidopsis* [25,26,27,28] and cassava [29], since the establishment of auxin gradients and auxin polar transport are essential for somatic embryo induction [26,30,31]. Conclusively, a combination of cytokinin and auxin could stimulate somatic embryo induction from embryo, cotyledon, and hypocotyl explants of *P. ostii* ‘Fengdan’.

However, different explants (embryo, cotyledon, and hypocotyl) had different responses on types of cytokinin and auxin. Better results of somatic embryogenesis from the zygotic embryos were obtained on the medium supplemented with both 0.5 mg·L^−1^ BA and 0.5 mg·L^−1^ 2,4-D or supplemented with both 0.5 mg·L^−1^ TDZ and 0.5 mg·L^−1^ 2,4-D, suggesting that 2,4-D plays an important role in the somatic embryo induction. Addition of an auxin (2,4-D) to the medium containing either BA or TDZ also effectively induced somatic embryos from the cotyledon explants of *P. ostii* ‘Fengdan’. Hypocotyls formed somatic embryos best on the MS medium supplemented with both 0.5 mg·L^−1^ TDZ and 0.5 mg·L^−1^ NAA. Abscisic acid (ABA) or a combination of BA and NAA could stimulate somatic embryogenesis from cotyledon explant of peony [18,32]. Phenylacetic acid (PAA) enhances somatic embryo induction from anther explant of peony [32], and a combination of BA and IBA induces somatic embryogenesis from shoot explant of Itoh peony [23]. The type of auxin and cytokinin and their optimum concentration for embryo induction have been proven to be different for different explants in many other plants [33,34]. The embryo formation on leaf explants is promoted by all the cytokinins (2IP, zeatin, kinetin, BA, and TDZ) in *Oncidium* [35]. In short, the most effective types of cytokinin and auxin vary with the explant. A combination of BA (a type of cytokinin) and NAA (a type of auxin) is reported to be effective in inducing somatic embryos and enhancing their development in many plants, such as *Rosa hybrida* [36] and *Carica papaya* [37], which was also fully demonstrated in *P. ostii* ‘Fengdan’ in our findings. Besides, concentration ratio of cytokinin (BA) and auxin (NAA) affected results a lot. The ratio of the explants with embryo induction and number of embryos appeared to be a single peak curve with the increase of concentration ratio of BA and NAA in *P. ostii* ‘Fengdan’ and the peak appeared at 3:1, which was also much better than other types of cytokinin and auxin combinations. Somatic embryos were successfully induced from the cotyledon explants of herbaceous peony (*P. lactiflora*) using the MS medium containing 3.0 mg·L^−1^ BA, 1.0 mg·L^−1^ NAA, and 1.0 mg·L^−1^ GA_3_ [18]. The induction ratio and differentiation frequency of somatic embryos also vary with concentration ratio of cytokinin and auxin in *Sapindus mukorossi* Gaertn. [38], *Eleusine coracana* [39], and *Coriandrum sativum* [40]. Conclusively, BA (as a kind of cytokinin) and NAA (as a kind of auxin) were effective for somatic embryo induction from embryo, cotyledon, and hypocotyl explants of *P. ostii* ‘Fengdan’ and most appropriate concentration were 3.0 mg·L^−1^ and 1.0 mg·L^−1^, respectively.

Significant differences for embryogenic potential were also observed within the explants of *P. ostii* ‘Fengdan’. The embryogenic potential was the greatest in zygotic embryos, followed by cotyledons and hypocotyls. Similar results were also found in other species. The best induction ratio of somatic embryos and the greatest number of embryos are also obtained from the zygotic embryos, which are significantly higher than those from cotyledon explants in *Murraya koenigii* [41]. Leaves and cotyledons also have a significantly better embryogenic response compared to hypocotyls in eggplant [33]. Callus induced from immature seeds are superior to those from hypocotyls or young leaves in the somatic embryogenesis of *Gentiana straminea* [42]. Besides, the effective basal media for embryo induction were different for different explants. The MS medium was better at inducing somatic embryos from the zygotic embryo and hypocotyl explants, while the WPM was better at inducing somatic embryos from the cotyledon explants. The maximum induction efficiency of embryos is also obtained on the MS medium with the hypocotyl explants of *Glycyrrhiza glabra* [43]. Somatic embryogenesis is efficiently induced from the zygotic embryos of *Beta vulgaris* on the MS medium [44]. Therefore, the best explant for somatic embryogenesis of *P. ostii* ‘Fengdan’ was embryo among three kinds of explants. The appropriate basal medium for embryo and hypocotyl explant of *P. ostii* ‘Fengdan’ was MS medium and better basal medium for cotyledon explant was WPM.

For the whole process of somatic embryogenesis from embryo, cotyledon, and hypocotyl explant of *P. ostii* ‘Fengdan’, compact callus was produced firstly and then changed to embryogenic callus with globular shape, roughness surface, and soft texture. Subsequently, somatic embryos in globular stage with smooth surface and compact structure were produced from those embryogenic calli. There were four stages, including globular stage, heart-shaped stage, torpedo stage, and cotyledonary stage, in the development period of embryos in *P. ostii* ‘Fengdan’. Similar stages are also detected in *Hypoxis hemerocallidea* [45], *Coriandrum sativum* [40], and *Citrullus lanatus* Thunb. [46]. Our results show that those somatic embryos induced from embryo, cotyledon, and hypocotyl explants of *P. ostii* ‘Fengdan’ developed well into mature embryos on their respective basal media supplemented with both 3.0 mg·L^−1^ BA and 1.0 mg·L^−1^ NAA. Furthermore, a combination of cytokinin and auxin, especially BA and NAA, enhanced induction and development of those somatic embryos. It is reported that BA combined with other PGRs promote the induction and development of somatic embryos in many other plants [47,48,49]. The growth medium containing both 3.0 mg·L^−1^ BA and 1.0 mg·L^−1^ NAA also enhances the development of somatic embryos in *P. lactiflora* [18]. 

## 5. Conclusions

Somatic embryogenesis was enhanced by a combination of cytokinin and auxin, especially by a combination of 3.0 mg·L^−1^ BA and 1.0 mg·L^−1^ NAA. The ratio of explants with embryo induction from the zygotic embryo and hypocotyl explants was the greatest on the MS medium supplemented with both 3.0 mg·L^−1^ BA and 1.0 mg·L^−1^ NAA, while that from the cotyledon explants was the greatest on the WPM supplemented with both 3.0 mg·L^−1^ BA and 1.0 mg·L^−1^ NAA. Zygotic embryos were the optimal explant for somatic embryo induction, followed by cotyledons and hypocotyls. These induced somatic embryos developed well into mature embryos on their respective basal media supplemented with both 3.0 mg·L^−1^ BA and 1.0 mg·L^−1^ NAA. Conclusively, the whole protocols were efficient for somatic embryogenesis from embryo, cotyledon, and hypocotyl explant of *P. ostii* ‘Fengdan’.

## Figures and Tables

**Table 1:** Effect of the basal medium and explant type on callus induction after 1 month of culture.

Explant	Medium	PGR Combination (mg·L^−1^)	Explant with Callus Induction (%)
Embryo	MS medium	3.0 BA + 1.0 NAA	0.86 ± 0.07a^z^
Embryo	WPM	3.0 BA + 1.0 NAA	0.85 ± 0.05a
Cotyledon	MS medium	3.0 BA + 1.0 NAA	0.53 ± 0.10b
Cotyledon	WPM	3.0 BA + 1.0 NAA	0.56 ± 0.07b
Hypocotyl	MS medium	3.0 BA + 1.0 NAA	0.80 ± 0.06a
Hypocotyl	WPM	3.0 BA + 1.0 NAA	0.82 ± 0.09a

The PGR combination used in this experiment is 3.0 mg·L^−1^ BA and 1.0 mg·L^−1^ NAA. ^z^ Separation by Duncan’s multiple range test at *p* ≤ 0.05.

## Supplementary Materials

The following are available online at https://www.mdpi.com/2223-7747/9/1/3/s1: Figure S1. Effect of the basal medium and concentration of BA and NAA on callus induction from the zygotic embryo. Scale bar is 1.0 mm. Unit of plant growth regulator (PGR) concentration is in mg·L^−1^. The data were recorded 1 month later; Figure S2. Effect of the basal medium and explant type on callus induction. The PGR combination used in this experiment is 3.0 mg·L^−1^ BA and 1.0 mg·L^−1^ NAA. Scale bar is 1.0 mm.
