# Supplementary material for: Enhanced Somatic Embryo Induction of a Tree Peony, Paeonia ostii ‘Fengdan’, by a Combination of 6-benzylaminopurine (BA) and 1-naphthylacetic Acid (NAA)
Source: Plants (Basel). 2019 Dec 18;9(1):3. doi: 10.3390/plants9010003 (PMC7020195; doi:10.3390/plants9010003)
Supplement: Supplementary file 1 [file plants-09-00003-s001.pdf]

Supplemental Figures

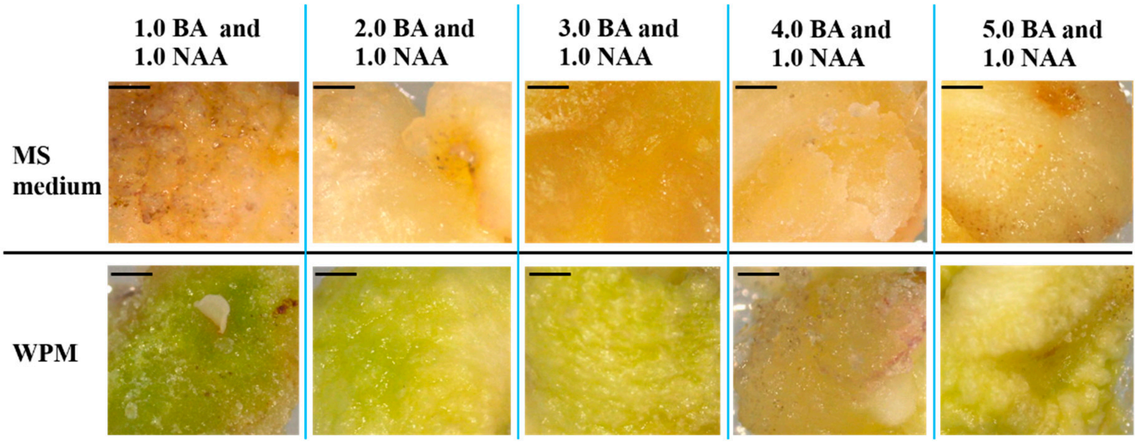

Figure S1. Effect of the basal medium and concentration of BA and NAA on callus induction from the zygotic embryo explant. Scale bar, 1.0 mm. Unit of PGR concentration is in mg·L^{-1}. The data was recorded 1 month later.

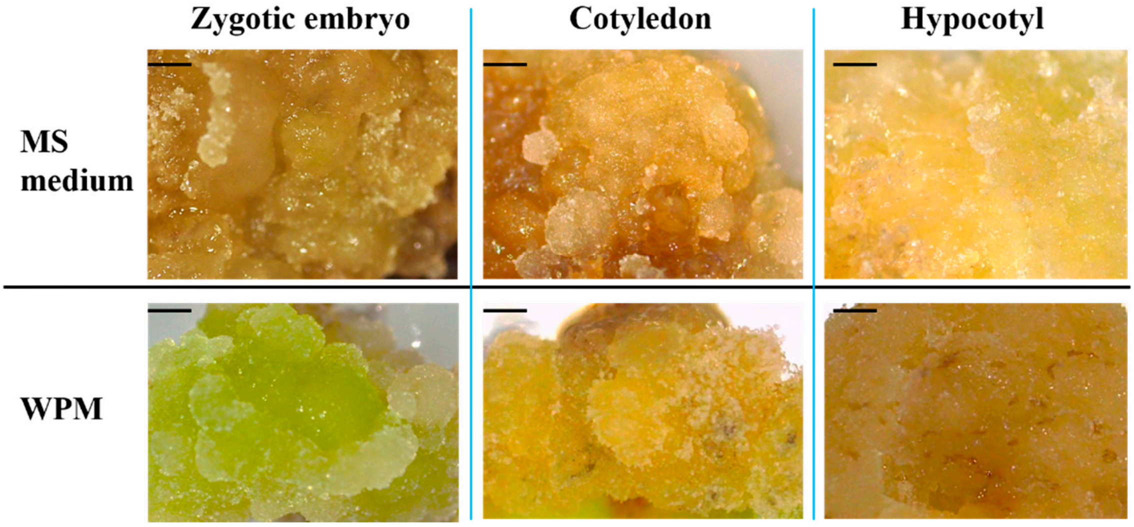

Figure S2. Effect of the basal medium and explant type on callus induction. The PGR combination used in this experiment is 3.0 mg·L^{-1} BA and 1.0 mg·L^{-1} NAA. Scale bar, 1.0 mm.
